# Degradable polymer bone adhesives

**DOI:** 10.1016/j.fmre.2023.11.023

**Published:** 2024-02-29

**Authors:** Zijian Bao, Ran Yang, Binggang Chen, Shifang Luan

**Affiliations:** a State Key Laboratory of Polymer Physics and Chemistry, Changchun Institute of Applied Chemistry, Chinese Academy of Sciences, Changchun 130022, China; b School of Applied Chemistry and Engineering, University of Science and Technology of China, Hefei 230026, China

**Keywords:** Bone adhesive, Biodegradable materials, Strong adhesion, Bone repair, Bone fracture

## Abstract

Highly comminuted fractures and bone defects pose a significant challenge for orthopedic surgery. Current surgical procedures commonly rely on metal implants (such as bone plates, nails and pins) for fracture internal and external fixations, but they are likely to result in problems, such as stress shielding and poor bone healing. Bone adhesive represents an attractive alternative for the treatment of fracture. The ideal bone adhesive should satisfy several performance requirements, including high adhesion strength for bone tissues, rapid in-situ curing in a physiological environment, good biocompatibility with no toxicity, degradability, and good stability *in vivo*. Among these requirements, degradability is a crucial characteristic of bone adhesives. This property enables the material to be easily removed without the need for surgery at a later stage, ensuring the regeneration of bone tissue without any hindrance. The degradation rate of bone adhesive varies depending on the application scenarios and tissues, ranging from weeks to years. Many bone adhesives are unable to guarantee degradability while achieving other necessary performances. Therefore, this article provides a detailed overview of the strategies to fabricate biodegradable polymer bone adhesives that can maintain high bulk and adhesion strength, biocompatibility and other properties. Finally, the current challenges in the clinical translation of bone adhesives and their future development directions are discussed.

## 1 Introduction

Each year, millions of people suffer from a variety of bone fractures due to infectious diseases, violent acts, traffic accidents, and sports injuries, which has resulted in the urgency of many surgical orthopedic treatments [1,2]. Internal and external fixations of bone fractures through implants such as bone plates, bone nails, or bone pins are now common methods clinically [3]. However, the utilization of these fixation devices may inevitably cause some negative effects, such as stress shielding, loss of mechanical properties of the bone over time, increased risk of implant infection, damage to surrounding tissues, and refracture. Moreover, bone plates and nails make it difficult to splice together small fragments of highly comminuted fractures or fractures with persistent bleeding [4,5]. This makes the treatment process extremely challenging and time-consuming, and may lead to serious and even life-threatening complications. More importantly, due to the limited degradation of implant materials such as metals and some nondegradable polymers, a second surgical intervention is needed to remove them after bone healing [6]. To address these issues, the introduction of bone adhesive biomaterials can provide an alternative method of fixing fractures [7].

Bone adhesive is a type of biomaterial that can bond different bone tissues and possesses many advantages over traditional fixation methods. Bone adhesives commonly have good contact with broken bone tissues and provide strong adhesion [8,9]. In addition, bone adhesive can easily adhere small bone fragments together and its lower hardness can reduce the stress shielding compared with metal implants. Its ease and simplicity of operation can also shorten the operating time [2,6]. Biodegradable bone adhesives, can achieve more effective bone healing without the need for removal through a second surgery [8,9]. All of these features make bone adhesives suitable for a variety of complex clinical applications, especially for the treatment of comminuted fractures. The ideal bone adhesives should meet the following performance requirements. First, the adhesion strength and cohesion strength should be high. Bone adhesives should provide good adhesion in dry and wet environments, and the cohesion strength should match the mechanical properties of the bone tissues. The mechanical load applied to the adhesive will drive crack propagation, resulting in the failure of the adhesive in the form of main cohesion [10]. High adhesion and cohesion strength provide a stable and reliable mechanical environment to prevent fracture [4]. Second, bone adhesives should have a degradation rate compatible with bone healing [3,6]. If the degradation rate of bone adhesives is too fast, it usually causes higher tissue toxicity or even ineffective stabilization, resulting in bone slippage or osteonecrosis. Conversely, if the degradation rate of the bone adhesives is too slow, it may prevent bone healing and cannot be resorbed or replaced by new bone. Moreover, the presence of nondegradable fragments may cause further serious complications [12]. Therefore, the bone adhesives should ideally be replaced by bone tissue after degradation and the degradation products should not interfere in bone remodeling. Third, bone adhesives should have rapid curing performance, and the curing reaction should be capable of occurring in a physiological environment. Fourth, bone adhesives should have good stability, the excessive swelling of the bone adhesive *in vivo* could reduce the adhesion strength and lead to peel failure [11], so the bone adhesives should maintain a low swelling state. Finally, the bone adhesives must be biocompatible and not generate excessive heat during curing, and the degradation products must be nontoxic and capable of being metabolized [13].

Due to the advantages of bone adhesives, many complex clinical scenarios of fractures in joint areas and highly comminuted fractures place very high expectations on them. Moreover, to avoid additional risks arising from secondary surgery, the biodegradable design of bone adhesive materials is particularly important, making it a key aspect in ensuring safety in biomedicine. To this end, the biodegradable materials used to fabricate bone adhesives in recent years are summarized in this paper, and the design strategies of polymers to achieve degradability and strong bone adhesion are described in detail. Although some materials may not be directly used in bone tissue at present, their degradation strategies *in vivo* should still be used for reference. Finally, the challenges of current bone adhesive materials in clinical transformation are also discussed.

## 2 Approaches to achieving biodegradable bone adhesives

Several mechanisms of degradation for polymer chains exist in organisms, including hydrolysis, enzymatic degradation, redox degradation, and pH-responsive degradation [11,[14], [15], [16], [17]. For materials applied *in vivo*, degradability is a critical safety issue. The degradation rate of tissue adhesives is considered depending on the specific application scenarios and tissues, and the time scale can range from several weeks to years [12]. The degradation process of the adhesives is jointly affected by the physical, chemical and biological interactions of the tissue microenvironment. In parallel, the geometry and molecular weight of the adhesive matrix also influence the rate and mechanism of degradation. In addition, the toxicity and metabolic pathways of the degradation products of the adhesives determine the final biocompatibility of the material. Polymer degradation products include nondegradable cross-linked fragments, cleaved molecular chains, low molecular weight polymer fragments and small molecule compounds [13]. These substances are generally excreted through the kidneys. The excretion molecular weight limit of the human kidney is 40–60 kDa [18,19]. Fragments with larger molecular weights may need metabolic clearance by hepatic uptake. For some degradable polymers, the degradation products are toxic or induce adverse reactions, making them unsuitable for use *in vivo* [20]. The following section mainly discusses the approaches to designing and constructing the biodegradable bone adhesives from three aspects: using biodegradable polymers as the main component, introducing biodegradable cross-linkers, and inserting biodegradable sites in the polymer backbone.

### 2.1 Bone adhesives with a biodegradable main component

According to the material types of the main components, bone adhesives can be divided into natural and synthetic adhesives.

#### 2.1.1 Natural adhesive*s*

Natural polymers have been widely used as tissue adhesives [21], due to their biodegradability and good biocompatibility. However, due to their rigid molecular chain structure, natural polymers are generally mechanically brittle. Additionally, some natural substances may carry a potential risk of spreading disease [22]. Natural adhesives can be classified into protein-based and polysaccharide-based bone adhesives based on their material sources. Protein-based adhesives mainly include fibrin glue, gelatin and albumin-based adhesives, which can be obtained by separation from human blood or animal tissues. Protein-based adhesives can usually act directly on tissues, while some require to be modified with reactive groups such as aldehydes and N-hydroxysuccinimide (NHS) esters. Adhesives based on polysaccharide composition include chitosan, alginate, dextran, and hyaluronic acid. Some polysaccharides are nondegradable *in vivo* and require chemical modification to degrade them hydrolytically [8,^13^,^17^,23].

Enzymatic degradation is the primary degradation pathway for protein and polysaccharide-based adhesives [14]. Natural materials are usually degraded by lysozyme or specific enzymes such as collagenase, gelatinase and hyaluronidase [24]. However, some natural polysaccharides such as dextran and alginate cannot be degraded *in vivo* due to the lack of corresponding endogenous enzymes in humans. To make them degradable for use under physiological conditions, these polysaccharides need to be reduced with low molecular weight or the polymer backbone chemically modified with structurally unstable sensitive sites [12]. For example, alginate was introduced into readily hydrolyzable linkages in its polymer chains *via* partial oxidation to achieve degradability *in vivo* [23]. Moreover, the synergistic action of multiple enzymes can lead to a faster degradation process. For example, gelatin can be rapidly degraded by gelatinases (MMPs-2, MMPs-9) and histone proteases [10,13].

**Fig. 1:**
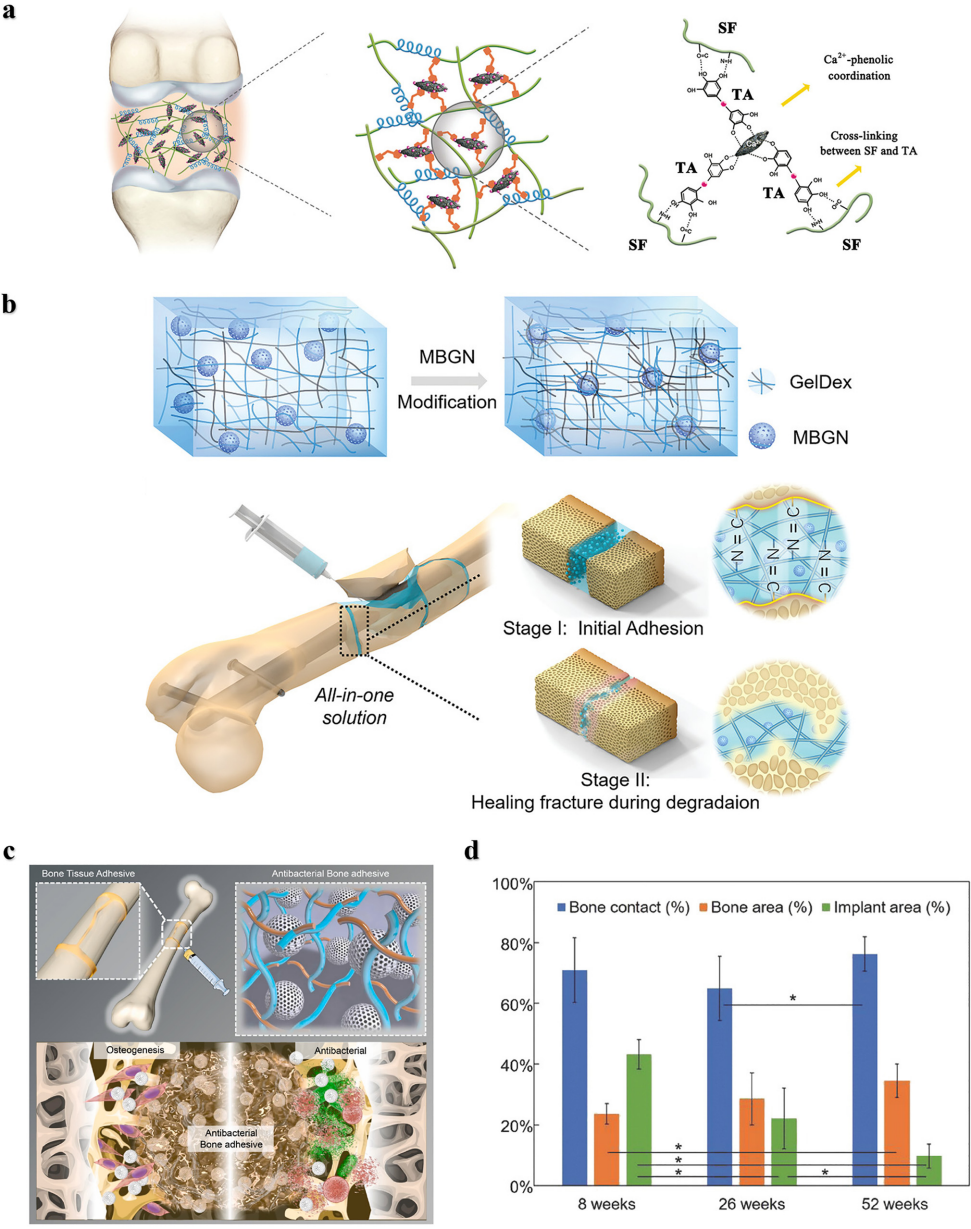
**Nature-derived Organic-inorganic strong bone adhesives.** (a) The components and interactions of SF@TA@HA: the nucleophilic-phenol interaction and calcium-phenol coordination of TA with SF and HA respectively endow it with high toughness and adhesion strength [25]. (b) Demonstration of the inorganic-organic strengthening of the adhesive via MBGN and GelDex, and bone glue as an all-in-one tool to flexibly adhere comminuted fragments [26]. (c) Multifunctional bone adhesive with anti-infection and osteogenic activity by doping porous nanobioactive glass loaded with vancomycin in modified gelatin and oxidized starch [27]. (d) Percentages of bone contact, bone area, and implant area over the 8–52 week period [28].

Although natural polymers are commonly used as tissue adhesives in fields such as hemostasis, dressing, wound closure, drug delivery, soft tissue repair, and biomedical devices, their poor mechanical strength and adhesion in wet environments make them unsuitable for hard tissue treatment, such as bone. Recently, inorganic bone‒promoting active nanoparticles combined with natural polymers to construct inorganic‒organic strong bone adhesives for fracture treatment have been widely studied. For example, *Yang* et al. prepared a hierarchically strong biomimetic bone adhesive SF@TA@HA using the interaction of silk fibroin (SF) with tannic acid (TA) and the coordination between tannic acid and hydroxyapatite (HA) to form a nanofiber structure [25]. The nucleophilic-phenol interaction and calcium-phenol coordination interaction in tannic acid endow the adhesive with strong adhesion, and the adhesion strength to wet bone reached 922 kPa and 607 kPa to wet bone covered with blood, respectively. The nanofiber structure mimicked the natural glue filaments between mineralized collagen fibers in bone and exhibited better toughness than polymethacrylate (Fig. 1a). *Chen* et al*.* constructed an injectable and reversibly adherent bone adhesive by incorporating amino-modified bioactive glass nanoparticles (MBGN) into gelatin-oxidized dextran networks (GelDex) [26]. The active dynamic covalent bond between the amine group and aldehyde group improved the dispersion stability of the nanoparticles, and the mechanical performances were also significantly enhanced due to the existence of highly dense dynamic covalent bonds (Fig. 1b). This bone adhesive can effectively fix the comminuted fracture fragments of the radius in rabbits and can promote bone regeneration and healing along with degradation. *Hu* et al*.* developed a multifunctional bone adhesive with anti-infection and osteogenic activity by doping porous nanobioactive glass loaded with vancomycin in modified gelatin and oxidized starch [27]. In addition to the Schiff base effect formed by gelatin and oxidized starch, the mesoporous bioactive glass acted as the cross-linker, further improving the mechanical performance and adhesion strength (Fig. 1c). *Gall* et al*.* prepared a new bone adhesive based on phosphoserine and tetracalcium phosphate that exhibited strong adhesion in wet environments and high compressive strength. After implantation in the femoral defects in rabbits, sustained osseointegration and osteoconductivity were demonstrated within 52 weeks (Fig. 1d) [28].

Although natural materials have good degradability and biocompatibility, their low bulk strength limits their application as bone adhesives [8,22]. Researchers have explored various strategies to improve the mechanical performances of natural materials, including the fabrication of covalent cross-linked double-network structures [25,^26^,29], and the incorporation of inorganic particles or organic fiber additives into natural polymers [27,29]. Despite the structural support provided by these methods, the natural materials still undergo cohesive failure when applied in load-bearing areas such as bone adhesion. Therefore, natural adhesives are mainly used in soft tissue adhesion. In contrast, synthetic adhesives can provide high-strength adhesion for bone tissues. Thus, many researchers are focusing on developing biodegradable synthetic bone adhesives.

#### 2.1.2 Synthetic adhesives

Polymethylmethacrylate (PMMA) and cyanoacrylates (CA) are the original synthetic materials used as bone adhesives [2,^8^,30]. PMMA-based adhesives are widely used as fillers for bone defects or as fixators for implants. PMMA connects bone tissue together primarily by mechanical interlocking, its low viscosity prior to solidification allows it to penetrate irregular interstices in the surface of the bone tissue by pressurization of the polymer, which enhances the contact area with the bone by anchoring [31]. Because its adhesion mechanism does not involve the interaction with bone tissue on the molecular scale, PMMA is not strictly a good choice as a bone adhesive. Furthermore, PMMA adhesive has disadvantages such as low adhesion strength in wet environments, thermal effect during polymerization leading to thermal necrosis of bone tissue, volume shrinkage, and poor compatibility with bone [9]. Various strategies have been adopted to overcome the above problems, including chemical surface modification, the addition of inorganic particles to improve mechanical strength, and the pretreatment of bone adherend. However, even with attempts to improve the degradation performance of PMMA adhesives by using biodegradable polymers to copolymerize with them, the nonbiodegradability of PMMA remains a major challenge [32]. CA adhesives can polymerize rapidly in a wet environment and achieve strong bone tissue adhesion. However, rapid polymerization in a short time releases a large amount of heat, leading to cell necrosis and tissue damage at the site of action [8,33]. In addition, the degradability of CA is relatively poor, and toxicity of the degradation products of polycyanoacrylate limits their clinical application. The degradation of methyl or ethyl cyanoacrylate with short alkyl chains would lead to the local accumulation of degradation products such as formaldehyde and cyanoacetate around tissues, which may cause severe inflammation and tissue necrosis. Furthermore, the brittle nature of cured cyanoacrylate adhesives makes them unsuitable for active and long-term applications [2,^8^,13]. To address the limitations of CA and PMMA adhesives in terms of poor biocompatibility, *in vivo* nondegradability/degradation toxicity and poor mechanical properties, a series of biodegradable and biocompatible tough adhesives have been explored and developed.

**Fig. 2:**
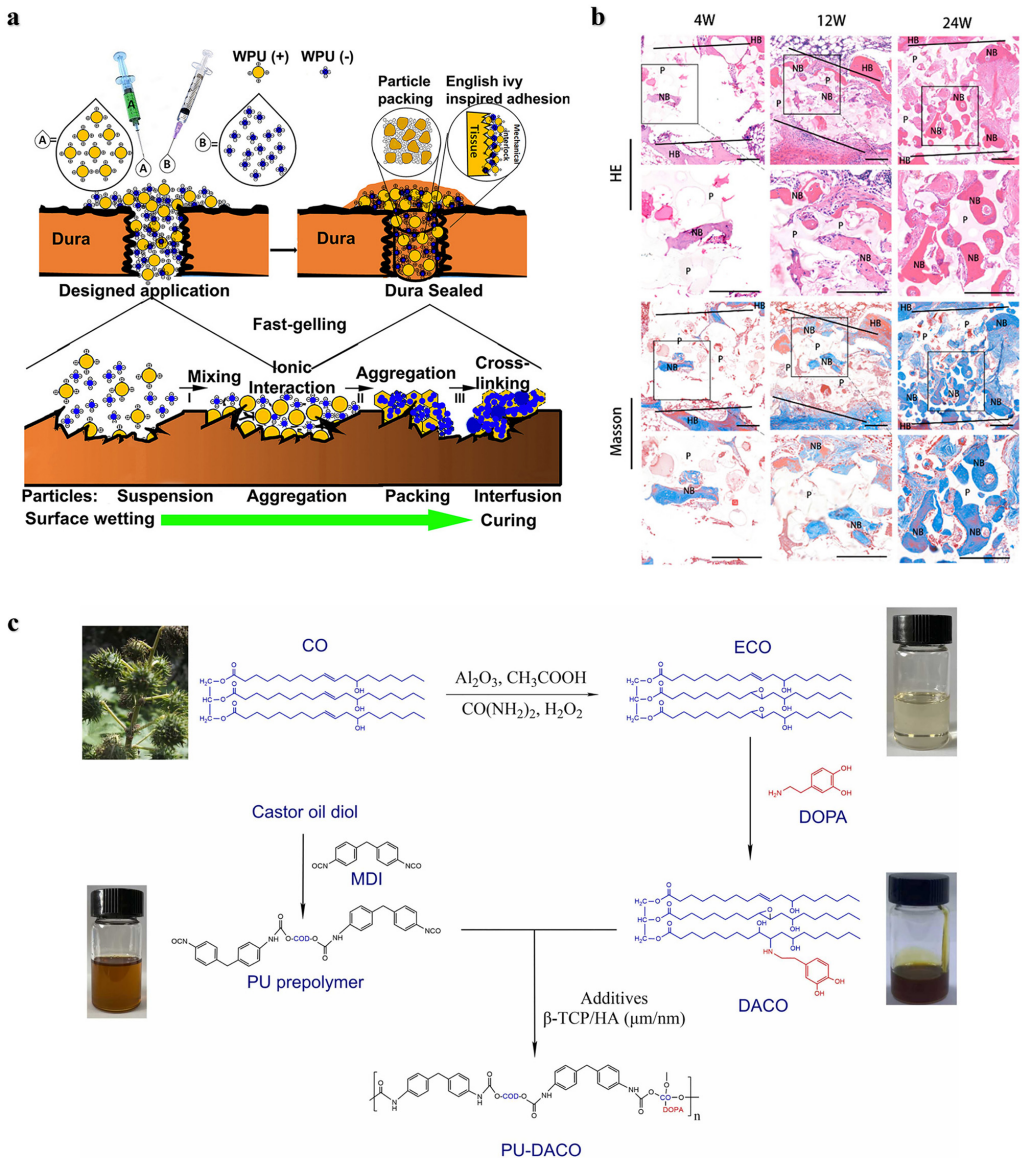
**Bone adhesives based on PU.** (a) Schematic representation of the adhesion mechanism of a two-component PU adhesive composed of positively charged A and negatively charged B nanodispersions [41]. (b) Histological analysis of transverse bone sections of PUA at 4, 12 and 24 weeks. NB: new bone, P: polymer, HB: host bone [42]. (c) The structures and synthetic routes of DACO and PU-DACO [43].

*Polyurethane*: Polyurethanes (PU) have shown promise as bone tissue adhesives due to the high reactivity of cyanates (NCO) with nucleophilic groups such as amines and hydroxyl groups present in biological tissues, which allows them to provide high adhesion strength. PU adhesives are biodegradable, can be degraded *in vivo* by hydrolysis and enzymatic hydrolysis (cholesterol esterase, elastase, proteinase K and lipase), and their degradation products are usually nontoxic and include polyol, lysine and carbon dioxide [13,^34^,35]. In addition, the mechanical properties and degradability of PU adhesives can be regulated by selecting polyisocyanates and polyols with different structures. The effects of different monomers, functional group ratios, and catalyst concentrations on the reactivity, biocompatibility, degradation rate, degradation mechanism, and mechanical strength of PU have been explored [36], [37], [38]. For example, two-component lysine-derived polyurethane has been investigated as an injectable biomaterial, and new bone formation was observed 6 weeks later in the metaphysis bone defect of the distal femur in rabbits [39]. *Becker* et al. prepared a phosphate-functionalized poly (ester-urea) copolymer as a degradable bone adhesive [40]. The interaction between the phosphoric acid on the polymer and the positive charge on the bone tissue promotes adhesion, which contributed to an adhesion strength of 439 kPa on bovine bone. However, exothermic heat during the reaction and unreacted monomer toxicity may cause damage to the tissue. Moreover, the PU material swells excessively after absorbing water, which leads to adhesion failure. *Liu* et al*.* prepared a tissue adhesive consisting of a two-component PU nanoparticle and counterions that resulted in a strong mechanical interlock between the adhesive and tissue interface through ionic cross-linking [41]. The penetration and stacking of different sizes of nanoparticles on the tissue surface and the ionic cross-linking between nanoparticles jointly enhance the adhesion strength. In addition, the PU nanoparticles formed a highly packed structure, giving this adhesive a higher mechanical strength (Fig. 2a). *Zheng* et al. developed a porous polyurethane bone adhesive (PUA) with enhanced adhesion strength and mechanical strength by adding polyisocyanate and β-tricalcium phosphate [42]. The adhesion strength of this bone adhesive was twice that of PMMA bone cement used in clinical practice. The results in a rabbit ilium transection bone model showed that the porous structure of PUA can promote cell penetration and new bone regeneration (Fig. 2b). This group then tried a two-component polyurethane (PU-DACO) made from dopamine-modified castor oil (DACO) to incorporate hydroxyapatite to improve bone compatibility and mechanical strength, with a 580 kPa adhesion strength in a wet environment (Fig. 2c) [43]. In two other studies, a multifunctional injectable polyurethane adhesive with aliphatic isocyanates and polyethylene glycol as the polymer backbone and cyclodextrin and gentamicin as cross-linkers and antibacterial agents, was investigated for use in hard tissue adhesion and sternal closure surgery [44]. The adhesive exhibited fast curing, high antimicrobial and strong adhesion and biodegradable absorption. Moreover, this PU adhesive can assist traditional wire loops for rapid sternal fixation and prevent postoperative sternal displacement [38]. In addition, the efficacy and stability of castor oil-based polyurethane adhesives were demonstrated in a subsequent randomized clinical trial in 29 patients who used the adhesive to assist sternal closure, with no adverse events or complications reported at early and after 12 months [45].

**Fig. 3:**
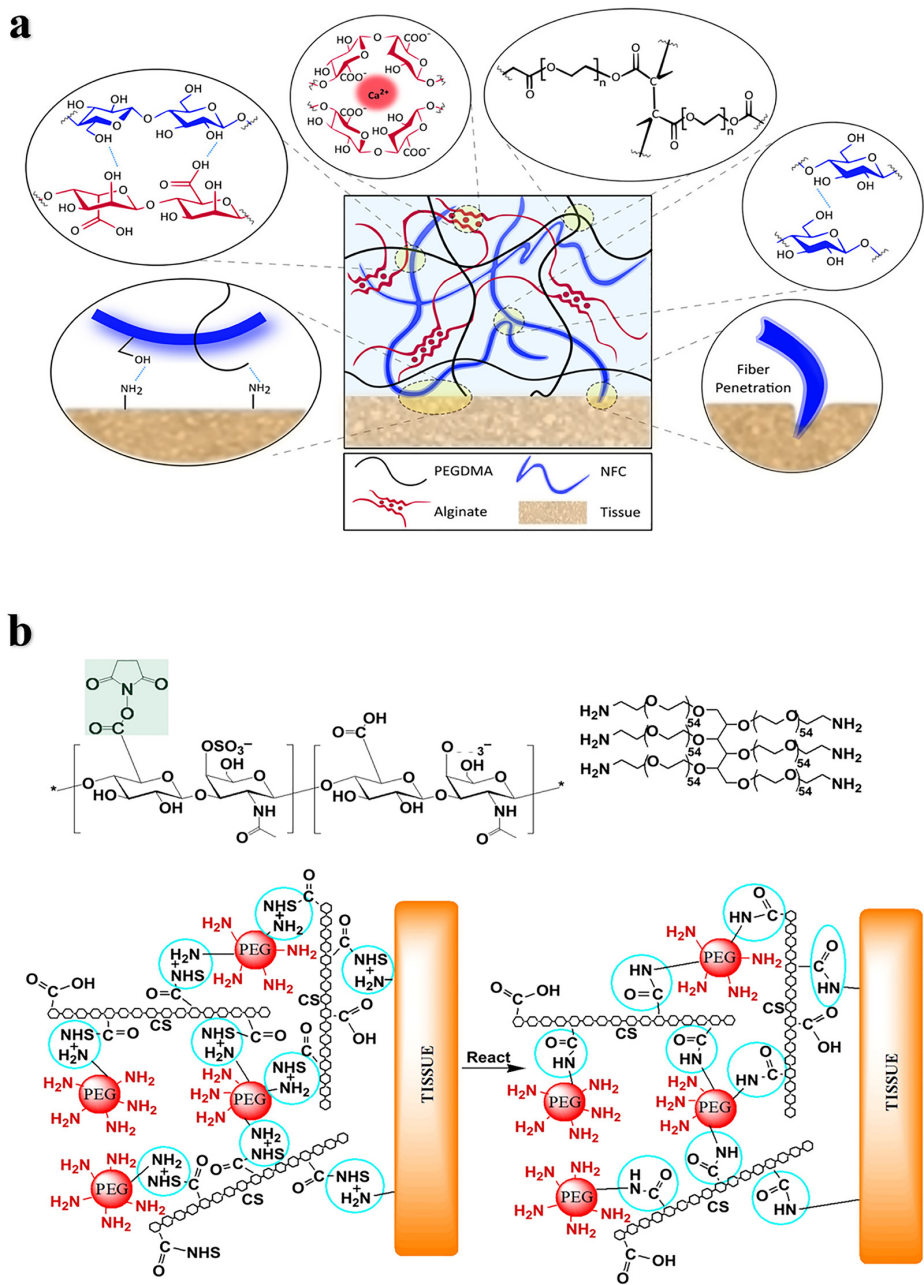
**Bone adhesives based on PEG.** (a) Schematic diagram of a hydrogel adhesive: The dissipative matrix consists of covalently cross-linked PEGDMA and ionic cross-linked natural alginate reinforced by cellulose fibers (NFC) [49]. (b) Structure composition and adhesion mechanism of CS-PEG [50].

*Polyethylene glycol*: Polyethylene glycol (PEG) is a biocompatible, water-soluble, nonimmunogenic and easily modified polymer that has been widely used in tissue adhesives. PEG is nondegradable and usually needs to be modified with degradable functional groups or copolymerized with degradable polymers [4,^46^,47]. Modified PEG is often used as a tissue adhesive in combination with natural materials such as polysaccharides or proteins. For example, a double cross-linked network hydrogel adhesive based on 8-arm PEG-amine with oxidized alginate methacrylic acid, modulated the mechanical performance and adhesion strength by varying the degree of oxidation of the alginate, and the adhesive strength was higher than that of fibrin adhesive [48]. *Pioletti* et al*.* designed a nanofiber-toughened double-crosslinked network hydrogel adhesive, which consists of an interpenetrating network formed by covalently cross-linked polyethylene glycol dimethacrylate (PEGDMA) and ionic cross-linked alginate (Fig. 3a) [49]. This bioadhesive can effectively resist the propagation of interface cracks and firmly adhere to cartilage and meniscus tissue, showing adhesion strength up to 130 kPa. *Strehin* et al*.* prepared NHS esterified chondroitin sulfate crosslinked with 6-arm PEG- (NH_2_) _6_ to form an adhesive hydrogel (CS-PEG) [50], and the carboxyl group activated by NHS could covalently link with the amine on the tissue surface to achieve high adhesion strength (Fig. 3b). The mechanical and swelling properties of CS-PEG can be tuned by changing the gel conditions such as pH, humidity and the proportion of components. CS-PEG can be hydrolyzed or enzymatically degraded with a weak inflammatory response. In addition to biocompatibility, the adhesion strength of CS-PEG to cartilage tissue is nearly 10 times higher than that of fibrin glue. The adhesive has been studied for the repair of cartilage tissue. For example, *Sharma* et al*.* combined chondroitin sulfate with PEGDA through a covalent bond and adhered to cartilage tissue at the joint defect to promote cartilage repair [51], it was crosslinked by light-initiated polymer solution and gelled after smearing *in situ* in a few minutes. They conducted a clinical study for this hydrogel adhesive-mediated cartilage repair in 18 patients. Fifteen cases of microfractures were treated with the PEG adhesive, and three cases without treatment were used as the control groups. The results of magnetic resonance imaging (MRI) showed that the filling rate of cartilage in the biomaterial implant was higher than that in the control group over time. However, due to the small sample size, these results do not fully prove the cartilage repair effect of the material, and further evaluation of these patients and a larger patient population is needed.

**Fig. 4:**
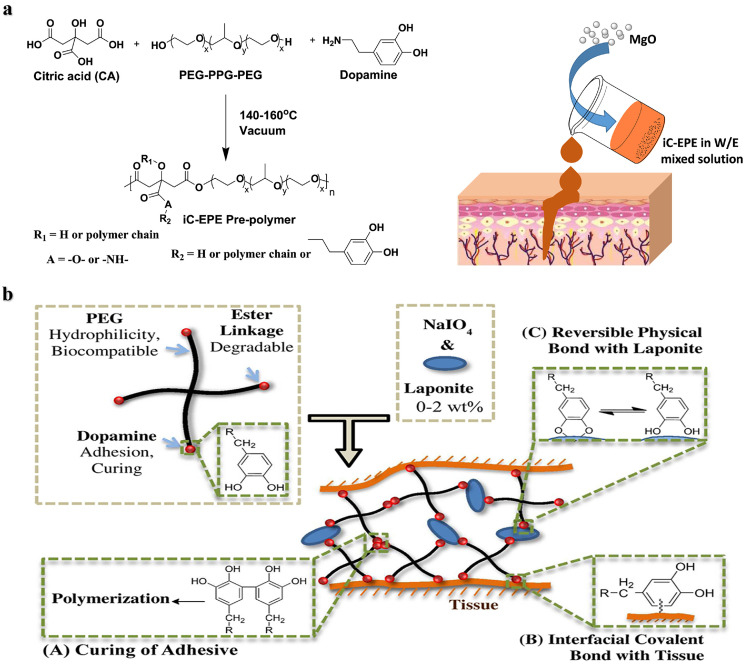
**Catechol-functionalized PEG adhesives**. (a) Synthesis of iC-EPE prepolymer, and magnesium oxide (MgO) serves both as a crosslinker and composite filler to form injectable citrate-based mussel-inspired bioadhesives [54]. (b) Structure of dopamine-functionalized four-arm PEG nanocomposite adhesives: curing, interfacial interactions and reversible physical crosslinking [56].

PEG is also often combined with biologically inspired functional molecules as bone adhesives, such as the catechol functional group DOPA [52]. *Yang* et al*.* [53] obtained an injectable bone adhesive, iCMBA/HA, by cross-linking DOPA and citrate-modified PEG with hydroxyapatite, which solidified within 4 min and showed good osteoconductivity. The release of citrate accompanied by the degradation of bone adhesive can promote the mineralization of human mesenchymal stem cells and new bone formation. In the rabbit model of comminuted fracture, the formation of neovascularization and highly organized bone was demonstrated. In addition, *Yang* et al*.* prepared a prepolymer iC-EPE with PEG-PPG-PEG triblock copolymer, citric acid, and dopamine. The iC-EPE was developed to be an injectable bioadhesive with magnesia oxide (MgO) particles as cross-linking agents and fillers [54], it finally exhibited a tensile strength of 1–4.5 MPa and an adhesive strength of 125 kPa, and the existence of magnesium ions and citrate endow it with application prospects in hard tissue regeneration (Fig. 4a). *Lu* et al*.* used functionalized DOPA and PEGDA crosslinked with poly(ethylene glycol) fumarate to enhance the attachment and proliferation of osteoblasts and wet adhesion to prevent displacement of the implanted bone defect site [52]. Hyperbranched polymers based on PEGDA, pentaerythritol tetraacrylate and dopamine were prepared by Michael addition reaction as tissue adhesives showing strong adhesion to bone tissues [55]. *Lee* et al*.* [56] reported a tissue adhesive hydrogel composed of dopamine-modified 4-arm PEG ester and nanosilicate, where the strong interactions of nanosilicate and dopamine shortened the curing time and enhanced the mechanical and adhesion performances (Fig. 4b). The degradation product orthosilicate was shown to promote the differentiation of osteoblasts *in vitro*.

*Polyester*: Aliphatic polyesters such as polylactide (PLA), poly(ε-caprolactone) (PCL), poly(lactide-*co*-glycolide) (PLGA) and polyglycolide (PGA), are widely used as scaffolds for bone regeneration in bone tissue engineering due to their controllable degradability, good biocompatibility, cell affinity, and certain mechanical properties. The degradation mechanism of polyester mainly involves hydrolysis and enzymatic degradation, and its degradation products are generally considered to be biocompatible. Experimental results evaluating the biocompatibility of the polyester material poly-l-lactide (PLLA), demonstrated minimal cytotoxicity after 7 days of incubation. These findings suggest that PLLA exhibits favorable biocompatibility [57], [58], [59]. However, acidic degradation products at the site of action may cause local inflammation and cytotoxicity [2,^13^,60]. Pure polyester adhesives usually have low adhesion strength, and isocyanate and acrylate modifications are generally needed to allow them to have good tissue adhesion performance [27,[61], [62], [63]. For example, a PCL-based adhesive combined with unsaturated acrylates was functionalized with different ratios of isocyanates. After 1 min of UV irradiation, the adhesive cured rapidly. By adjusting the content of the isocyanate group, it showed different swelling rates, degradation rates and adhesive strengths [64]. In another study, two adhesives synthesized by the reaction of PCL with isophorone diisocyanate (IPD) and hexamethylene diisocyanate (HDI) showed low swelling behavior. PCL-IPD exhibited much a higher adhesion strength than PCL-HDI, which is probably due to presence of the more free isocyanate groups that could covalently link with the surface of tissues [65]. Lactic acid-based oligomers were modified using three different acrylate monomers by Gil et al. [66]. The oligomers underwent rapid polymerization within three minutes through free radical polymerization in the presence of a photoinitiator. These three acrylate-modified adhesives exhibited varying rates of swelling and degradation, the evaluation of their biocompatibility and bioadhesion properties indicated that these adhesives can effectively maintain wound closure and facilitate repair [67]. As bone adhesive, polyester hydrolyzes over time in a wet environment, and the adhesion strength may decrease significantly along with severe cytotoxicity. Therefore, it is still a challenge to develop a polyester adhesive with high adhesion strength [68].

**Fig. 5:**
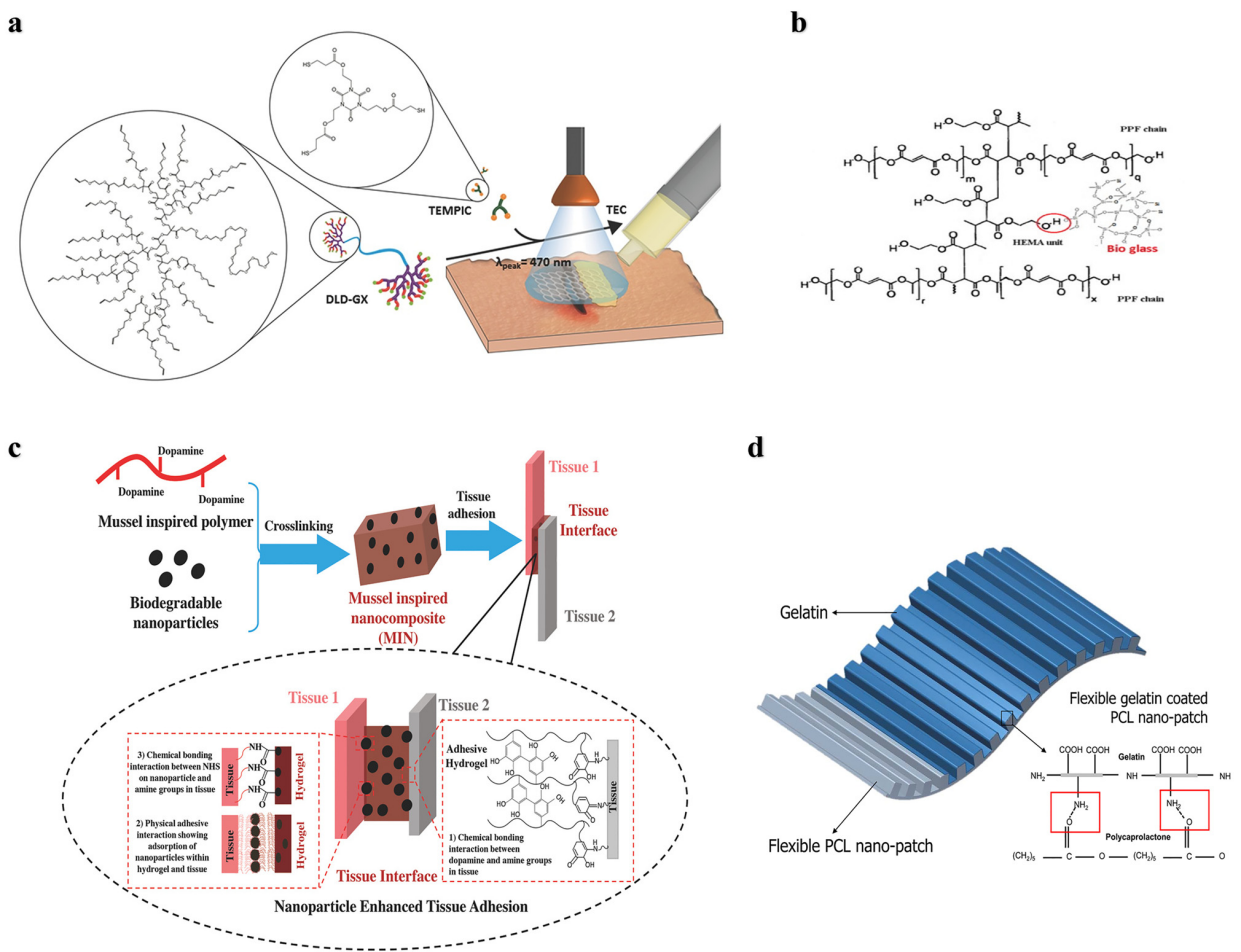
**Strategies for improving adhesion and mechanical properties of polyester adhesives**. (a) Hyperbranched polyester was cured by photoinitiation of the thiol-ene click reaction to create a highly crosslinked tissue adhesive [69]. (b) Nanobioactive glass-reinforced propylene fumarate (PPF) bone adhesives [71]. (c) NHS-modified PLGA biodegradable nanoparticles with dopamine-modified alginate to enhance tissue adhesion through three interactions between tissues and nanocomposite adhesive [72]. (d) Schematic illustration of flexible gelatin coated PCL nanopatch [73].

To address the problem of the low adhesion strength of polyester materials, researchers have constructed various strategies. First, a dendritic network structure was constructed to improve the mechanical properties of the polyester adhesives. For example, a hyperbranched polyester based on 2,2-dihydroxymethylpropionic acid was cured by photoinitiation of a thiol-ene click reaction to create a highly crosslinked tissue adhesive with low swelling and good adhesion in a wet environment (Fig. 5a) [69]. Second, inorganic particles such as bioactive ceramics combined with degradable polyesters have been extensively developed. The enhanced mechanical properties, adjustable biodegradability, and biological activity of these composites make them attractive as bone adhesives. For example, a mixture of tricalcium phosphate, PGA and PCL bioadhesive reported by *Amyl* et al*.* overcame the low mechanical strength and brittleness of ceramics, achieving good mechanical properties, hydrophilicity and osteoconductivity. The incorporation of nanoparticles provides more options for bone tissue regeneration and bone repair [70]. Nanobioactive glass-reinforced propylene fumarate bone adhesives have been used in orthopedic surgery, and the addition of bioactive glass significantly enhanced the tensile strength, compressive strength, and modulus of the material, which obviously promoted cell proliferation and biomineralization (Fig. 5b) [71]. Third, adhesion performance in wet environments can be improved by introducing polyester nanoparticles as a reinforcing phase. For example, the hydrogel composed of NHS-modified PLGA nanoparticles and dopamine-modified alginate showed a higher adhesion strength due to the interaction of catechol groups and the nanoparticles as a bridge between the tissue and the hydrogel (Fig. 5c) [72]. In addition, researchers have developed many other methods to enhance the adhesion performances of polyester adhesives. For example, *Kim* et al. prepared a PCL patch with a nanopatterned surface resembling an extracellular matrix structure, and then applied gelatin to the nanopatterned PCL patches to form biomedical patches [73]. Nanopatterning increased the surface area of the PCL, thereby enabling more gelatin to be coated on its surface (Fig. 5d), this effectively improved the hydrophobicity, adhesion and mechanical performance of PCL under the synergistic effect of nanotopographical pattern and gelatin coating, which was demonstrated by the high shear adhesive strength of the biopatch at 352.3 kPa under wet conditions. A two-component bone adhesive based on polylactic acid and methacrylate was used to immobilize a rabbit model of monocondylar distal femur osteotomy. The adhesive showed good short-term (84 days) biocompatibility without interfering with the fracture healing process [74]. In a subsequent long-term study of the sheep tibial lateral condyle osteotomy model over 6 months, this bone adhesive showed significant osteolysis and osteonecrosis without any signs of bone healing. These results may have been due to the uncontrolled degradation of the polymer, the reduction of the surrounding tissue pH by acidic degradation products and the highly crystalline fragments formed when PLA was degraded [75].

### 2.2 Bone adhesives with biodegradable crosslinkers

Linking polymer chains through degradable units or reversible chemical bonds is an attractive strategy to achieve material degradation. Using degradable crosslinkers allows polymers with different structures to be degraded at chain junctions. This strategy also enables the regulation of the polymer backbone and crosslinking density, resulting in material degradation at a suitable rate. At present, a series of cross-linkers with unstable functional groups have been designed and developed for constructing tissue adhesives. This strategy allows a variety of different crosslinkers to be obtained by changing the properties of the polymerizing groups and degradable functional groups. [10], [11], [12].

**Fig. 6:**
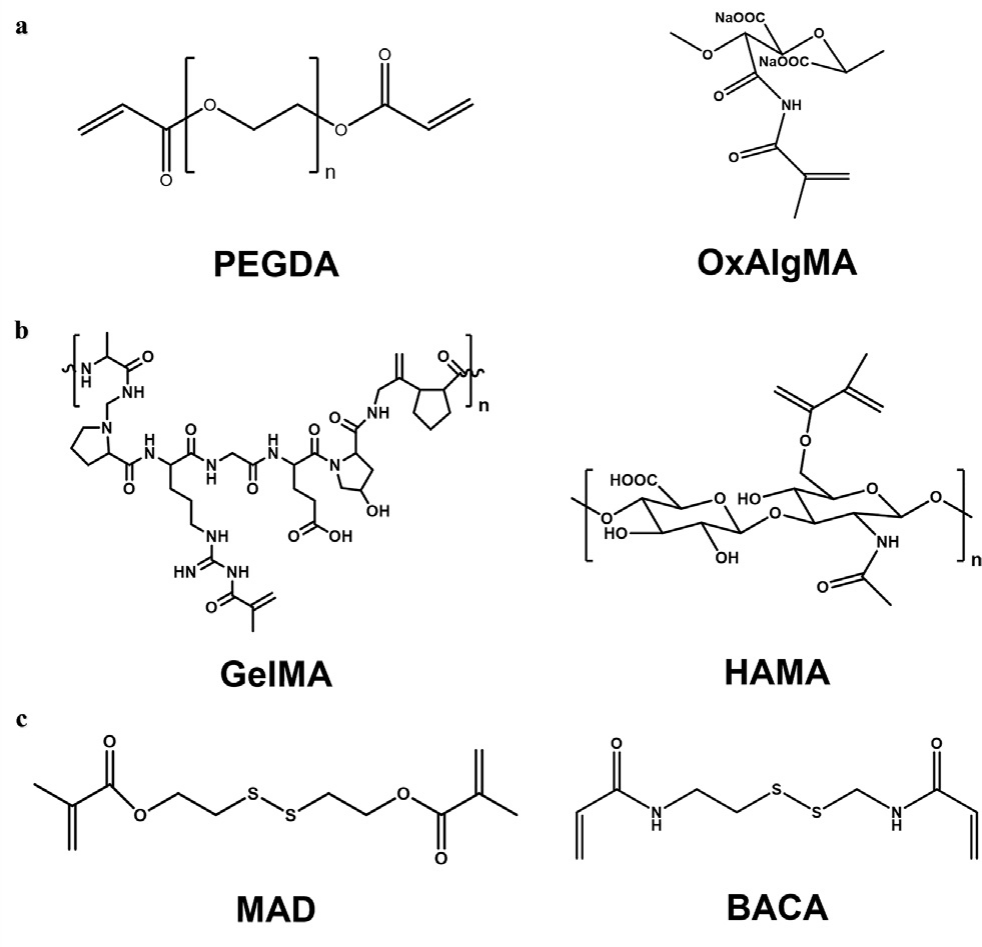
**Degradable crosslinkers:** (a) Hydrolyzed poly(ethylene glycol) diacrylate (PEGDA) and oxidized alginate methacrylate (OxAlgMA) [49,78]. (b) Enzyme-degraded gelatin methacrylate (GelMA) and hyaluronic acid methacrylate (HAMA) [76,78]. (c) Disulfide bonds- containing bis(2-methacryloxyethyl) disulfide (MAD) and N,N’-bis (acryloyl) cystamine (BACA) [12,79].

In general, there are some biodegradable units with different degradation behaviors, such as hydrolyzable poly(ethylene glycol) diacrylate (PEGDA), oxidized alginate methacrylate (OxAlgMA) (Fig. 6a), enzymatic degradable gelatin methacrylate (GelMA), and hyaluronic acid methacrylate (HAMA) (Fig. 6b) [49,[76], [77], [78]. PEGDA contains a nondegradable poly(ethylene glycol) molecular chain. Therefore, the molecular weight of poly(ethylene glycol) should not be too high, so as to maintain the degradability of PEGDA. Disulfide-containing compounds, such as bis(2-methacryloxyethyl) disulfide (MAD) and N,N’-bis (acryloyl) cystamine (BACA) and its derivatives, are also the universal biodegradable cross-linkers (Fig. 6c) [12,79]. Disulfide bonds act as dynamic covalent interactions that can be cleaved or rearranged in response to chemical or physical stimuli. *Wang* et al. prepared a hydrogel adhesive that cured *in situ* within 1 min using a disulfide-containing cross-linker and aldehyde-functionalized hyaluronic acid [80]. The introduction of disulfide bonds endowed the hydrogel system with self-healing properties, and enhanced its degradability *in vivo*. Furthermore, it could remove reactive oxygen species from the wound site and reduce oxidative stress-related damage. *Suo* et al*.* introduced the disulfide cross-linker BACA into the alginate-polyacrylamide double network to prepare a strong and degradable adhesive. The disulfide bond serves as a cross-linking point for polyacrylamide, allowing for disulfide-mercapto exchange interactions and thus degradation of the polyacrylamide network. The adhesive achieved strong bonding to different biological tissues and can withstand high tensile strength to cartilage tissue and over 160 mmHg of water pressure to lung tissue [79]. In addition, degradable junctions can be achieved by inserting degradable multifunctional molecules that have both initiator and monomer fragments, and such molecules can readily form degradable hyperbranched structures. A star polystyrene has been prepared by atom transfer radical polymerization (ATRP) with initiators containing disulfides [81]. The disulfide and ester bonds can be cleaved separately by different chemical methods, which endows the material with stepwise degradation properties along with physical and chemical changes. Such controllable and selective degradation of materials with diverse responses through the introduction of different unstable functional groups such as disulfide bonds or o-diol groups may hold great promise in bioadhesives.

**Fig. 7:**
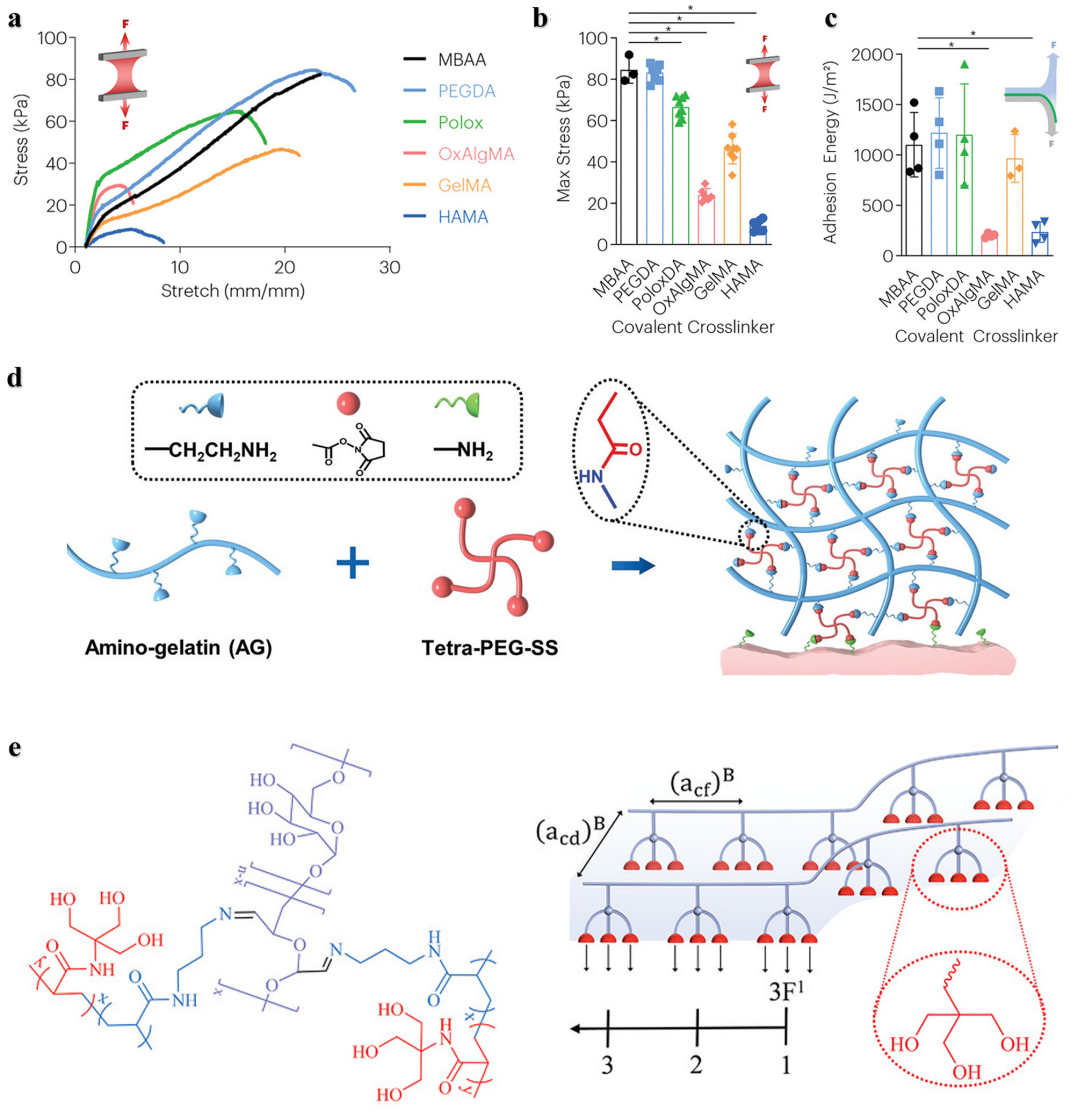
**Strategies to improve the mechanical properties and stability of degradable crosslinker-crosslinked bone adhesives.** The stress-stretch curve (a), maximum stress (b) and adhesion energy (c) of hydrogel adhesives with different crosslinkers, with N,N′-methylene bis(acrylamide) (MBAA, nondegradable crosslinker) as the control group [78]. (d) The crosslinking and adhesion mechanism of the hydrogel adhesive AG-PEG [82]. (e) The p(APMA-co-THMA) crosslinked with dextran-CHO to form a biodegradable adhesive hydrogel with high a density of hydrogen bonds by introducing “triple hydrogen bonding clusters (THBC)” [83].

Although these crosslinkers can achieve polymer degradation, not all units are suitable, and the introduction of some degradable units may reduce the adhesive strength and mechanical performance of the materials. For example, *Mooney* et al. investigated the effects of a range of crosslinkers degraded through hydrolysis or enzymatic degradation on the mechanical toughness, adhesion performance, degradation rate, and biocompatibility of hydrogel adhesives. Compared with those adhesives containing nondegradable crosslinkers, the mechanical properties and adhesion strength of the hydrogel adhesives with degradable crosslinkers other than PEGDA and poloxamer diacrylate (PoloxDA), were reduced, especially those with OxAlgMA and HAMA (Fig. 7a-c) [78]. An effective solution is to improve the mechanical properties of the materials by increasing the crosslinking density with an appropriate amount. Increasing the crosslinking density also shortens the curing time of the adhesive, but too short of a time will cause uneven crosslinking. For example, a hydrogel adhesive AG-PEG based on an amino modified gelatin (AG) and a four-arm polyethylene glycol succinimide succinate (Tetra-PEG-SS) exhibited high toughness and adhesion strength, biodegradability, and rapid self-gelation (Fig. 7d) [82]. The introduction of amino groups improved the cross-linking density and the mechanical performance of the adhesive, while allowing it to self-gel in approximately 1 min. Moreover, the active ester group of succinimide can form a covalent bond with the amino groups on the tissue, which provides strong chemical adhesion. Since many biological applications such as tissue adhesives require a high density of functional groups, the bifunctional copolymers described above can form hyperbranched structures that have also attracted much attention [12]. In another study, *Ma* et al*.* reported a hydrogel adhesive with a high density of hydrogen bonds by introducing “triple hydrogen bonding clusters (THBC)” into the side group of the polymer chain (Fig. 7e). The hydrogel was fabricated by copolymerization of N-[tris(hydroxymethyl) methyl] acrylamide (THMA) and N-(3-aminopropyl) methacrylamide hydrochloride (APMA), and the three hydroxyl groups carried by THMA endowed the adhesive with strong tissue adhesion even without any chemical reaction. To make the hydrogel stable and biodegradable in the long term, they used oxidized glucan as a crosslinker [83]. *Kim* et al*.* reported a dopamine modified acrylate terminated tri(ethylene glycol) diacrylate-dopamine crosslinker (TDC), which was used to construct a double crosslinked hydrogel adhesive by photopolymerization with acrylic acid. The mechanical properties of the hydrogel mainly depend on the content of TDC. With increasing of TDC content, the hydrogen bonding, oxidation and Schiff base reaction between dopamine and functional groups on the surface of tissues were enhanced, thereby improving the tensile and adhesion strength of the hydrogel [84].

### 2.3 The backbone of polymer adhesives introducing degradable sites

Endowing tissue adhesives with degradability can also start with the preparation of polymer materials, this involves introducing unstable or degradable units into the polymer backbone. Only a small number of degradable links are needed to achieve a substantial reduction in the polymer molecular weight. In cases where degradable units are uniformly distributed in the polymer chain, a single degradable unit can reduce the molecular weight of the polymer chain by half. Therefore, the polymer chain can be degraded to the range of renal metabolism by introducing multiple degradable units or combining unstable functional groups. For example, a degradable junction was placed between two polymer chain segments by using a bifunctional initiator containing a degradable unit, such as disulfide bonds, Diels-Alder adducts and hemiacetal esters [81,^85^,86]. A bifunctional initiator synthesized with 2-bromopropionic acid and bis(2-hydroxyethyl) disulfide can be used to obtain polymers by ATRP. Disulfide bonds in the polymer chains can be cleaved by the reaction of dithiothreitol or glutathione [87]. Patrickios et al. synthesized a degradable bifunctional group initiator with an acid-unstable hemiacetal group that can be effectively cleaved in the middle of the polymer chain in which it is located [86].

**Fig. 8:**
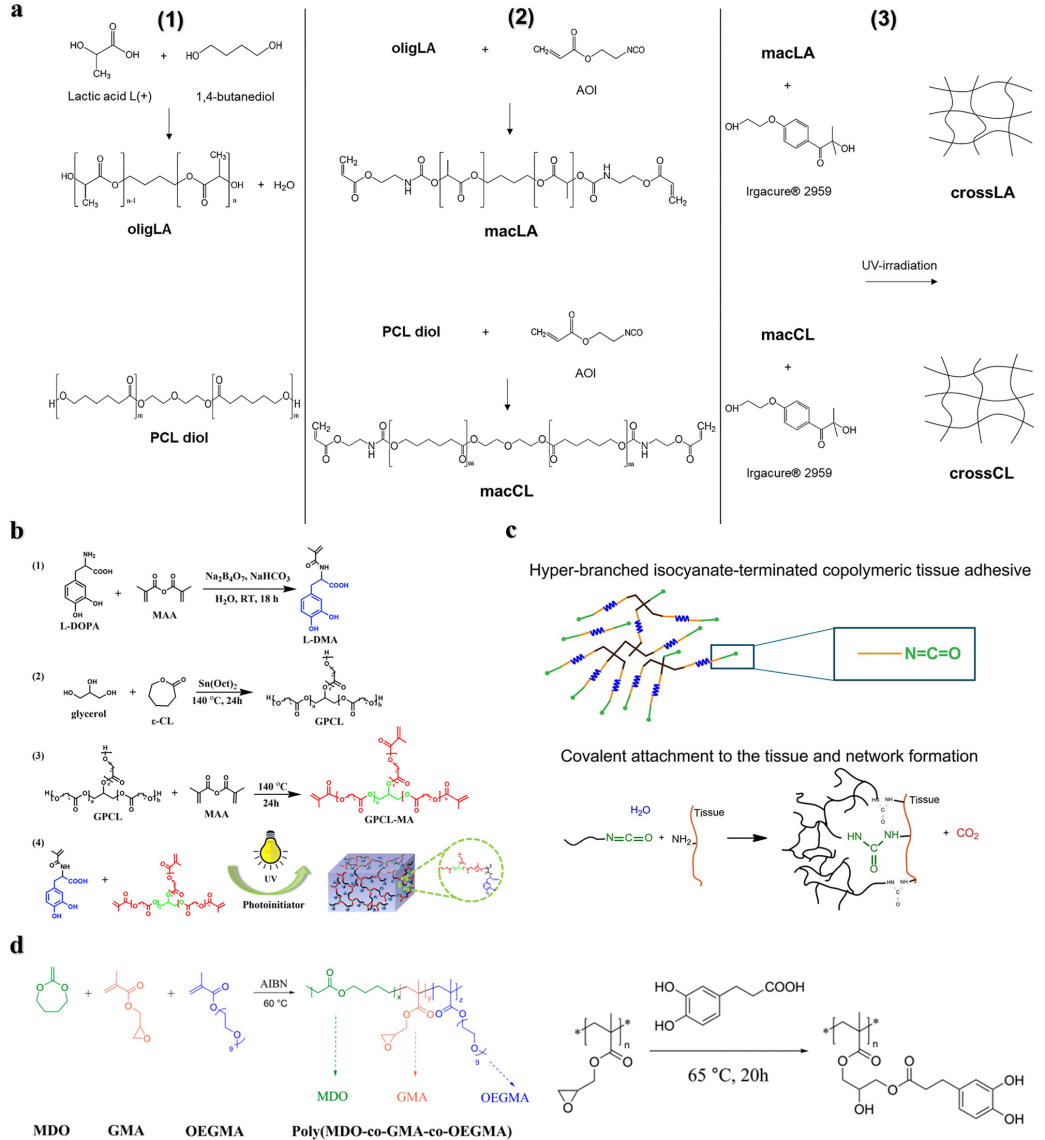
**Strategies for introducing degradable units into the polymer backbone**. (a) Preparation process of biodegradable adhesives based on UV light-initiated cross-linking of lactic acid and polycaprolactone oligomers [88]. (b) Degradable mussel-like adhesive prepared by copolymerizing glycerol and methacrylic anhydride-modified PCL units with 3,4-dihydroxyphenyl-l-alanine acrylamide (L-DMA) molecular chains via UV polymerization [89]. (c) Degradable hyperbranched tissue adhesive process prepared by introducing polyethylene glycol esters into the polymer backbone. Yellow: poly(trimethylene carbonate), blue: PEG, black: citric acid, green: hexamethylene diisocyanate [90]. (d) Synthesis of poly (MDO-co-GMA-co-OEGMA) via rROP and its catechol-functionalized reaction process [68].

Introducing bifunctional precursors or adding degradable molecules to produce multisegment degradable polymers is also one of the ways to achieve degradation. This strategy has been demonstrated in dithiol-terminated polymers, molecules/oligomers containing anhydrides, thioesters, esters, and enzymatically degradable polypeptide sequences [13,^16^,52]. For example, *Ferreira* et al*.* designed two types of 2-isocyanoethyl acrylate end-sealing functionalized materials with oligomers of lactic acid and polycaprolactone as the matrix, and prepared two types of UV photo-triggered cross-linking adhesives (Fig. 8a). The lactic acid adhesive showed better hydrolysis, biocompatibility and antibacterial properties in a simulated physiological environment than the caprolactone adhesive [88]. Bai et al. prepared mussel-mimetic adhesives by copolymerizing glycerol and methacrylic anhydride modified PCL units with 3, 4-dihydroxyphenyl-l-alanine acrylamide (L-DMA) by UV polymerization (Fig. 8b). Incorporation of PCL enhanced the biocompatibility and biodegradability of the adhesive. When the amount of l-DMA was 60 mol%, the adhesion strength to porcine skin reached 2.13 MPa [89]. *Grijpma* et al*.* used PEG as an initiator for the ring-opening polymerization of trimethylene carbonate to prepare a degradable chain segment. They then polycondensed it with citric acid to obtain a hyperbranched copolymer, and finally its terminal group was functionalized with isocyanates to prepare a tissue adhesive (Fig. 8c). The adhesive can be used as a meniscus repair agent, and its adhesion strength to bovine meniscus is higher than that of fibrin glue used in clinical practice [90]. Significant degradation can be achieved by preparing multiblock degradable polymers through macromolecular conjugation or by adding degradable units to the polymer backbone. Radical ring-opening polymerization (rROP) of cyclic monomers is a method to insert degradable groups into the polymer backbone and achieve degradation. The rROP presents significant advantages over other polymerization methods, including a wide variety of selective polymeric monomers, achieving reversible deactivating radical polymerization, and synthesizing controllable degradable polymers. Moreover, the absence of metal catalysts makes it promising in the biomedical field [12,91]. Several classes of cyclic monomers that can perform rROP have been developed, such as cyclic ketene acetals (CKA), thionolactones, and macrocyclic monomers, among them, CKA monomers are the most studied [12]. The CKA monomers can copolymerize with the acrylates to form the backbone degradable polymers through rROP, which can be implemented *in situ* at room temperature by a redox initiator and is suitable for the development of tissue adhesive materials [92]. For example, *Agarwal* et al*.* prepared a degradable copolymer poly (MDO-co-GMA-co-OEGMA) via the rROP of CKA monomer 2-methylene-1,3-dioxepane (MDO), glycidyl methacrylate (GMA), and oligo (ethylene glycol) methacrylate (OEGMA) and then functionalized it with catechol groups, it exhibited good adhesion performance on pig skin (Fig. 8d) [68]. In a separate study, Wang et al. enhanced the degradability of conventional bone PMMA cement by incorporating the MDO monomer to copolymerize with MMA through rROP. As a result, the obtained bone cement with MDO exhibited favorable biocompatibility and mechanical properties comparable to those of traditional PMMA bone cement [93]. Recently, Luan et al. proposed utilizing *in situ* rROP to fabricate robust and backbone-degradable tissue adhesive BDRAs. The BDRAs mainly consist of hydrophobic monomer MDO and hydrophilic acrylate comonomers, which ensures good wetting and penetration for different substrates, resulting in high adhesion strength. It was demonstrated that the adhesion strength of BDRA was up to 16 MPa for wet bone and 150 kPa for porcine skin. Furthermore, it was found that BDRAs could be effectively tuned by selecting different acrylate comonomers to regulate the degradation rate, mechanical properties, and setting time. The versatility allows BDRAs to adhere to both soft and hard tissues. This work provides a promising strategy for developing strong and degradable polymer bone adhesives [94].

## 3 The challenges of bone adhesives clinical transformation

Bone adhesives that can be rationally designed and specifically tailored to match bone tissues are better candidates for translation into the clinic. However, there are currently no commercial bone adhesive products available. A major challenge is to form sufficiently stable and strong adhesions for bone tissues. Currently available bone adhesives have difficulty obtaining and maintaining high adhesion strength in challenging clinical environments (especially wet environments) [3,^4^,6]. Moreover, fracture healing generally takes three months or more, so bone adhesives need to maintain the corresponding adhesion performance during this period. Another challenge in the clinical translation of bone adhesives is that they should not impede or disrupt any subsequent stages of fracture healing [8]. The complex process of secondary fracture healing requires the coordination of corresponding cells at various stages for long-term bone remodeling. Therefore, the level of mechanical support provided by bone adhesives to the fracture site and the appropriate degradation rate are the key factors determining the ultimate clinical performance of these adhesives. In addition, the blood supply to the fracture site may be reduced, resulting in slow or even nonunion. Therefore, it is crucial that the application of bone adhesive does not destroy blood vessels. Quantitative evaluation the efficacy of adhesion needed is extremely difficult. It depends on the method of adhesive application, the position of the implementation in the body, and the needed load-bearing strength. Finally, bone adhesion-related infections should be avoided as much as possible to avoid adverse effects on fracture healing [95], [96], [97].

Researchers should fully consider the clinical needs of bone adhesives and obtain predictable results through experiments. Effective measures should be taken to monitor the long-term performance of bone adhesives after implantation. Moreover, it is necessary to evaluate the impacts of the stability and degradation of bone adhesives *in vivo* on the adhesion performance, cohesive properties, and immune response of the materials. For example, it should be assessed whether the degradation products of adhesive materials may cause tissue inflammation. In addition, refined and standardized models should be developed to assess the properties of bone adhesive materials *in vivo* and *in vitro*. In general, the preparation of bone adhesives should be simple, and the adhesion operation should be achieved within the clinically needed time to minimize the duration of surgery in clinical application [9,^30^,98]. Moreover, developing new adhesive material systems and proposing practical design concepts are of paramount importance. These include synthesizing new chemical-structured materials, exploring novel preparation modes, optimizing physical and chemical properties, and investigating new mechanisms of tissue adhesion. Considering the clinical transformation of bone adhesives, it is crucial for researchers to collaborate with clinicians to reassess the design, development, and evaluation of bone adhesives [21], so that novel adhesives for targeting specific tissues can meet the desired performance requirements and address clinical needs. In other words, interdisciplinary collaboration will foster the emergence of new ideas, testing methods, and materials for bone repair.

## 4 Summary and prospects

In this review, we first summarize the performance requirements of bone adhesive materials and focus on the strategies for achieving *in vivo* degradation of bone adhesives. Natural adhesives have good biocompatibility, but their low adhesion strength and mechanical properties limit their clinical application. Synthetic adhesives can offer higher adhesion strength, but the biological toxicity after implantation as well as the inflammatory and immune reactions caused by material degradation products, cannot be ignored. Using degradable crosslinkers can maintain the stability of the material and impart degradability. However, this does not allow for complete degradation of the material, preventing bone tissue healing to some extent and potentially reducing the mechanical and adhesive properties. Some natural crosslinkers degrade too rapidly to provide effective mechanical support during the treatment period. Furthermore, only a small number of degradable sites introduced in the polymer backbone can achieve significant degradation of the material. Moreover, the uncontrollable sequence of degradable units in the polymer backbone can cause large differences in degradability, which may affect the stability of the material.

To date, there are still no commercial bone adhesives for fracture repair and trauma surgery. Constructing bone adhesives that meet performance requirements is a great challenge. Bone adhesives have great clinical potential, and clinical transformation products may be realized in the future.

## Declaration of competing interest

The authors declare that they have no conflicts of interest in this work.

## Appendix Supplementary materials

Image, application 1
