# Supplementary material for: Degradable polymer bone adhesives
Source: Fundam Res. 2024 Feb 29;5(2):782–95. doi: 10.1016/j.fmre.2023.11.023 (PMC11997572; doi:10.1016/j.fmre.2023.11.023)
Supplement: Supplementary file 1 [file mmc1.docx]

***Response to reviewers***

Sep. 15, 2023

*Journal: Fundamental Research*

*Manuscript ID:* *FMRE-D-23-00317*

*Title: Degradable polymer bone adhesives*

*Corresponding Authors: Shifang Luan, Binggang Chen*

**Dear Editor**

We appreciate your efforts in considering and reviewing our manuscript (FMRE-D-23-00317). The manuscript has been carefully revised according to editor’s and reviewer’s comments. The point-by-point responses to the reviewer comments are as follows. Should there be any questions or suggestions, please feel free to contact me.

**Referee #1**

**Question 1:** The abstract needs to be revised. Please incorporate some important degradation experimental dates obtained from the other researches to this part.

**Our Response:** Thank you for your kind suggestions. We have supplemented the abstract with the degradation data of bone adhesives according to different scenarios from other researches. The relevant details in manuscript (page1, line 20-22) are as follows:

‘*The degradation rate of bone adhesives is varying depending on the application scenario and tissues, ranging from weeks to years.’*

**Question 2:** The font size of some legends in the text was too small and should be made larger to make it more convenient for readers. Additionally, some image scales were not standardized and unified enough, please further improve to make them easier to understand.

**Our Response:** Thank you for your comments. To make it easier for readers to understand, we have increased the font size of the sub-plots in Figures 5, and 8, and corrected the scale of some images in the manuscript.

**Question 3:** References should refer to the latest literature more frequently, as well as improve the relevance and quantity of the literature. Besides, the format of the references should be verified again and should be consistent with the requirements of Fundamental Research.

**Our Response:** Thanks for your suggestion. We added some relevant literatures in the last three years. And we have carefully revised the reference format, including the journal titles, author, year, title of the work, and other publication information.

**Question 4:** This study was lack of sufficient biological experiments to accurately evaluate the biodegradable polymer bone adhesives. Suggesting supplementing more biological analysis (such as biocompatibility analysis) to better support your research objectives and also add color to the article. The following literature might provide some references: 10.1016/j.jallcom.2023.171526, 10.1016/j.colsurfb.2023.113512, 10.1016/j.nanoen.2020.104825.

**Our Response:** Thank you for your kind suggestions. We have added some biocompatibility analysis based on the above literatures in the polyester adhesives section of the manuscript. The relevant details in manuscript (page 11, line 346-348) are as follows:

*‘Experimental results evaluating the biocompatibility of polyester materials* *poly-l-lactide (PLLA), demonstrated minimal cytotoxicity after 5 and 7 days of incubation in vivo, respectively. These findings suggest that PLLA exhibits favorable biocompatibility [57-59].’*

**Referee #2**

**Question 1**: There are some language errors in this manuscript. For example, in Lines 19-20, Page 4, "as it cannot be removed by a second surgery in the later stage and should not hinder the bone tissue regeneration''; page 6, lines 98-100, "In parallel the geometry and molecular weight of the adhesive matrix also influence the rate and mechanism of degradation"; page 9, lines 209-211, "even with attempts to improve the degradation performance of PMMA adhesives by using biodegradable polymers copolymerized with it". The grammar faults exist in these sentences. Please check and revise the complete manuscript in terms of these errors.

**Our Response:** Thank you for these comments. We have made grammatical corrections to the above sentences as follows, and some other grammatical errors presented in the manuscript are also checked and revised.

*‘This property enables the material to be easily removed without the need for surgery at a later stage, ensuring the regeneration of bone tissue without any hindrance.’*

*‘In parallel, the geometry and molecular weight of the adhesive matrix also influence the rate and mechanism of degradation.’*

*‘However, even with attempts to improve the degradation performance of PMMA adhesives by using biodegradable polymers to copolymerize with it, the non-biodegradability of PMMA remains a big challenge.’*

**Question 2:** In 2.1.2 Synthetic adhesives, the isocyanate and acrylate modifications are generally required to allow polyester adhesives with good tissue adhesion performance. The authors did not further discuss the acrylate modifications in polyester adhesives. Please overview this part with relative examples.

**Our Response:** Thanks for your suggestion. We have added a discussion of acrylate modifications in polyester adhesives with examples. The relevant details in manuscript (page 12, line 360-366) are as follows:

*‘Lactic acid-based oligomers were modified using three different acrylate monomers by Gil et al [66]. The oligomers underwent rapid polymerization within three minutes through free radical polymerization in the presence of a photoinitiator. These three acrylate-modified adhesives exhibited varying rates of swelling and degradation. The evaluation of their biocompatibility and bio-adhesion properties indicated that these adhesives can effectively maintain wound closure and facilitate repair [67].’*

**Question 3:** In 2.2 Bone adhesive with biodegradable crosslinkers, the authors mentioned that the hydrolysable poly(ethylene glycol) diacrylate (PEGDA) and oxidized alginate methacrylate (OxAlgMA). Among them, the PEGDA comprises the nondegradable poly(ethylene glycol) macromolecular chain. Therefore, the molecular weight of PEG cannot be too high. Otherwise, it will affect the degradation of the PEGDA. Please explain this problem in the manuscript.

**Our Response:** Thank you for this comment. We have added an explanation about the problem of the PEGDA with the high-molecular weight PEG macromolecular chain would affect its degradation, in the relevant part of the manuscript (page 14,15, line 435-438).

*‘The PEGDA contains a non-degradable poly(ethylene glycol) molecular chain. Therefore, the molecular weight of the poly(ethylene glycol) should not be too high, so as to maintain the degradability of PEGDA.’*

**Question 4:** In 2.3 The backbone of polymer adhesives introducing the degradable sites, constructing the degradable polymer adhesives through radical ring-opening polymerization (rROP) the authors mentioned is a novel strategy. Are there any reported literatures currently using rROP to fabricate degradable bone adhesive? Please refer to the relevant literatures to give a discussion.

**Our Response:** We have searched for the relevant literatures on the preparation of biodegradable bone adhesives by rROP. As a result, there are no related literatures about using rROP to fabricate degradable bone adhesives. Here we give a discussion about an examples that using rROP to fabricate the degradable bone cement. The relevant details in manuscript (page 18, line 563-567) are as follows:

*‘In a separate study, Wang et al. enhanced the degradability of conventional bone PMMA cement by incorporating the MDO monomer to copolymerize with MMA through rROP. As a result, the obtained bone cement introducing MDO exhibited favorable biocompatibility and mechanical properties comparable to those of traditional PMMA bone cement [93].’*

**Question 5:** In the 3. The authors list some measures to overcome the challenges of bone clinical transformation, including monitoring the long-term performances of bone adhesive, evaluating the impacts of the degradation in vivo on adhesion performances, developing standardized models, and so forth. Based on these measures, it is more important to create new adhesive materials systems and propose practical design concepts, to meet the performance requirements of bone adhesives as far as possible. Please give a more detailed discussion about this part.

**Our Response:** Thank you for your helpful suggestions. We have supplemented a related discussion according to the suggestions. The relevant details in the manuscript (page 20, line 610-619) are follows:

*‘Moreover, developing new adhesive materials system and proposing practical design concepts are of paramount importance. These include synthesizing new chemical-structured materials, exploring novel preparing modes, optimizing the physical and chemical properties, and investigating new mechanisms of tissue adhesion. Considering the clinical transformation for bone adhesives, it is crucial for researchers to collaborate with clinicians to reassess the design, development, and evaluation of bone adhesives [21], so that the novel adhesives for targeting specific tissues can meet the desired performance requirements and address clinical needs. In a word, interdisciplinary collaboration will foster the emergence of fresh ideas, testing methods, and materials for bone repair.’*

**Question 6:** The recently published review or research articles should be discussed in the revision, for example, 10.1021/acsami.1c17434.

**Our Response:** According to your comment, we have cited and added a discussion of the relevant literature in the manuscript (page 2, line 42-44).

We really appreciate all your kind assessment and thoughtful reviewing comments. Thank you so much, and best regards.

Sincerely Yours,

Prof. Shifang Luan

State Key Laboratory of Polymer Physics and Chemistry

Changchun Institute of Applied Chemistry

Chinese Academy of Sciences

Renmin Street, Changchun 130022, P. R. China

Tel.: +86 431 85262159; fax: +86 431 85262109
